# Whole-Body MRI with Diffusion-Weighted Imaging in Bone Metastases: A Narrative Review

**DOI:** 10.3390/diagnostics8030045

**Published:** 2018-07-09

**Authors:** Alessandro Stecco, Alessandra Trisoglio, Eleonora Soligo, Sara Berardo, Lidiia Sukhovei, Alessandro Carriero

**Affiliations:** Ospedale Maggiore della Carità di Novara, Istituto di Radiodiagnostica ed Interventistica, Università del Piemonte Orientale, Amedeo Avogadro, Corso Giuseppe Mazzini 18, 28100 Novara, Italy; alessandratrisoglio@outlook.it (A.T.); eleonora.soligo@alice.it (E.S.); berardo.sara@gmail.com (S.B.); lid.sukhovei@gmail.com (L.S.); profcarriero@virgilio.it (A.C.)

**Keywords:** whole-body magnetic resonance imaging, diffusion-weighted imaging, bone metastasis, lung cancer, breast cancer, thyroid cancer, melanoma

## Abstract

Whole body magnetic resonance imaging (MRI) with diffusion-weighted imaging (WB-MRI-DWI) is currently emerging as a diagnostic technique in the evaluation of bone metastases from breast, prostate, lung, thyroid, and melanoma tumors. The most relevant articles regarding the detection of solid tumor bone metastases with MRI have been reviewed and cited. The imaging methods currently used in the detection of bone metastases are bone scintigraphy, computed tomography (CT), and positron emission tomography (PET/CT) with 2-deoxy-2-[fluorine-18] fluoro-d-glucose (18F-FDG PET/CT). WB-MRI-DWI allows qualitative and quantitative evaluation of focal lesions through signal intensity evaluation on DWI images and the reconstruction of the apparent diffusion coefficient (ADC) map. In prostate and breast cancer, WB-MRI-DWI is useful in assessing the response of bone lesions to therapy and to detecting early non-responders, while in lung cancer the method shows a similar sensitivity to 18F-FDG PET/CT in the detection of bone metastases. In bone metastases of thyroid tumors and melanoma, the WB-MRI-DWI shows a higher sensitivity when compared to 18F-FDG PET/CT. With a standardization of the WB-MRI-DWI protocol, this method seems to play an important role in the diagnosis of bone solid tumor metastases.

## 1. Introduction

Bone metastases cause a significant reduction in quality of life, producing pain and pathological fractures. Bone metastases are detectable in patients with advanced stages of breast, prostate, lung, and thyroid cancer, as well as melanoma. They are also minor entities in uterine and gastrointestinal tumors, as well as other types of neoplasms.

The most useful imaging techniques for the detection of bone marrow secondary lesions were bone scintigraphy (BS), contrast enhanced computed tomography (CE/CT), and positron emission tomography (PET/CT) with 2-deoxy-2-[fluorine-18] fluoro-d-glucose (18F-FDG PET/CT). A few decades ago, magnetic resonance imaging (MRI) began to be used in diagnostic practice; its use does not lead to ionization radiation exposure.

Diffusion-weighted imaging (DWI) is based on the evaluation of microscopic movements of water at the cellular level, providing quantitative (e.g., apparent diffusion coefficient (ADC)) and qualitative (e.g., signal intensity) information, which can be used to distinguish benign from malignant disorders. This pulse sequence is mandatory for oncologic imaging and in the specific field of distant metastases detection.

Recently, whole-body magnetic resonance arose as a new technique in assessing systemic disease and has been improved by the addition of DWI pulses (WB-MRI-DWI).

In our review, we concentrated on evaluating the role of WB-MRI-DWI and, particularly, the additional information provided by DWI sequences in the most common adult tumors which metastasize to bones.

## 2. Protocols and Pitfalls

Bone localizations, detected with WB-MRI-DWI, appear hypointense in T1-weighted images, hyperintense in fat-suppression sequences, and hyperintense in DWI with a low ADC value, due to the restriction of water and enhancement after contrast administration.

To view the appearance of bone metastases, a WB-MRI-DWI protocol should include these sequences: T1W, T2 with or without fat suppression, and DWI [1].

However, it is not always as easy as it seems. Metastases characteristics are not the same because the primary tumor and its secondary lesions have different microstructures. All secondary bone lesions could be divided into two main groups: osteoblastic and osteolytic. These do not always have the same appearance on DWI sequences which may be a problem, as shown in the study by Bauret et al. where sclerotic metastases treated with chemotherapy had a hypointense appearance on DWI due to low water content [2,3]. The solution seems to be a protocol which includes morphologic and functional sequences, including DIXON sequences that offer faster acquisition and a more homogenous suppression of fat [4].

The protocol might also include “whole spine” acquisition, as reported in previous literature, to evaluate all the vertebrae in the sagittal plane. In this way, it is possible to improve the accuracy of lesion localizations thanks to a higher spatial resolution, instead of a solely coronal plane. On the other hand, pathological fractures, vertebra collapses, and involvement of the medullary channel may be discovered in the sagittal plane. Sometimes a differential diagnosis between malignant lesions and other conditions could be very difficult, despite the application of DWI sequences. On T2-weighted sequences and on DWI, the hypersignal of bone marrow may induce false positive interpretations, such as for edema related to fractures, degenerative diseases, bone infarctions, infections, hemangiomas, isolated red bone marrow islands, and treated inactive lesions (T2-shine through). An ADC map can lead to a correct diagnosis. False negative results could be found in the evaluation of the skull due to the high signal of the adjacent brain [1,5].

## 3. Diffusion-Weighted Imaging (DWI) Sequences in Bone Metastases Detection

After the introduction of DWI sequences into the WB-MRI standard protocol, it has become easier to identify and differentiate benign bone marrow lesions from skeletal metastases [6].

### 3.1. Prostate Cancer

Prostate cancer (PC) is a common genitourinary malignancy which affects males and is the second most common cause of cancer mortality. Its incidence continues to rise in western countries, with the most frequent bone metastases located in the pelvis, spine, ribs, and femurs [7,8].

The diagnosis of PC is based on rise of prostate-specific antigen (PSA) serum values. Radiological imaging is necessary to distinguish local and systemic recurrence.

The Prostate Cancer Consensus Conference of 2017 reported the use of CT and scintigraphy for the detection of metastatic bone disease, although these methods of image analysis have low sensitivity and specificity. They mentioned the WB-MRI-DWI as a new method to improve the detection of bone metastases; it has an intrinsic bias in that there is an absence of a histopathologic control for each lesion and the absence of a gold standard to verify the real presence of tumoral cells [9].

Bone scintigraphy (BS) is considered a commonly used technique for suspected bone lesions. Nowadays, comparative studies of WB-MRI and PET/CT (with different radio tracers) have shown that their metastasis detection in advanced cancer is more accurate than that of BS.

PET/CT has been used with different tracers for the detection of prostate metastases, such as ^11^C-choline, ^18^F-fluorocholine, and ^18^F-Na fluoride. In particular, ^18^F-NaF PET/CT is a highly sensitive technique but, with regards to specificity, it is necessary to consider the tracer accumulation in inflammatory and degenerative bone diseases [10].

WB-MRI can identify skeletal involvement with higher sensitivity than BS and the same sensitivity as PET/CT, as Shen et al. demonstrated in his meta-analysis where the three techniques were compared [11].

Hengqing et al., in his region-based analysis, highlighted the importance of WB-MRI at the level of the spine and pelvis, which are the most frequent sites of bone metastases for prostate cancer; on the other hand, they demonstrated the weakness of the technique in some skeletal segments, such as its low sensitivity in the ribs, clavicles, and scapulae [12].

Moreover, the most important aspect is that quantitative DWI can provide clear categorization of the treatment response of bone metastases due to calculation of the ADC value. BS and PET/CT are able to identify only disease progression, as was demonstrated in two recent studies by Padhani et al. and Lecouvet et al. [13,14].

Some studies evaluated the advantages of using DWI in determining the treatment response of advanced prostate cancer. An example is that of Barchetti at al. who assessed the accuracy of DWI with different morphological sequences in detecting skeletal metastases in 152 patients who had undergone DWI for restaging after treatment. The MRI protocol, including sequences T1W, T2W, STIR, and DWI, showed a sensitivity of 99%, a specificity of 98%, a positive predictive value of 98%, a negative predictive value of 96%, and an accuracy of 98% for the identification of bone metastatic lesions [15].

The real limit of DWI is the absence of studies which report a standardized “gold standard” to verify the real presence of bone metastases. In literature, several studies used other diagnostic exams to verify the real presence of bone metastases, like CT, PET/CT, X-ray, or follow-up. However, no study used bone biopsy as a control [16,17,18].

The question must be asked: can WB-MRI with DWI replace BS for single-step detection of metastases in patients with high-risk prostate cancer? Lecouvet F. et al. asked this in his work in 2012. They answered “yes”, like Shen at al. in their 2014 metanalysis and Phadani et al. in their works [11,19].

**Figure 1:**
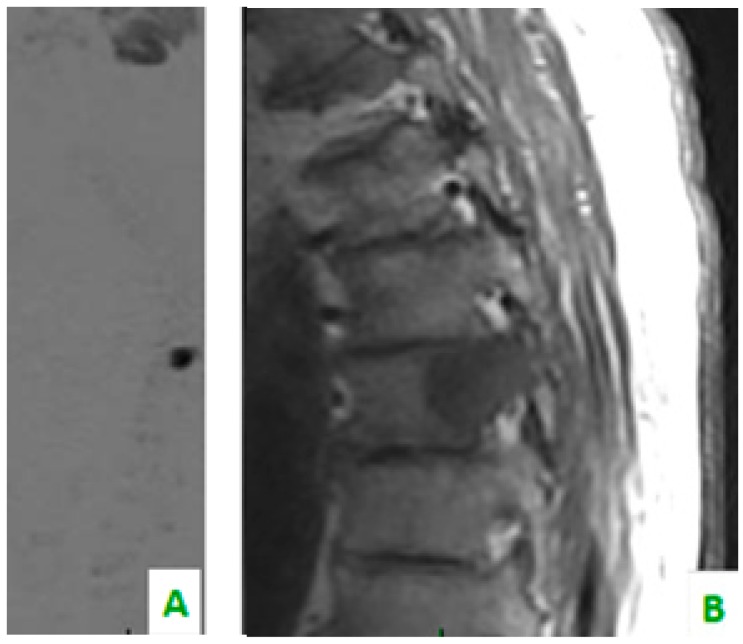
(**A**) Diffusion-weighted imaging (DWI) sequence in sagittal plane shows focal alteration of diffusivity. (**B**) The sagittal plane of TSE T1 shows, in the same level, a hypointense region of the dorsal soma related to the secondary localization of prostate cancer.

Despite the fact that there are numerous works that support the importance of WB-MRI-DWI for the identification of prostatic skeletal metastases in literature, this technique is not yet universally recognized, as evidenced in a recent work by Wieder et al. showing the superiority of ^11^C-choline PET/CT in detecting bone lesions (*p* = 0.02). In this work, however, no difference was reported in the detection of lymph node metastasis *(p* = 0.65) [20] (Figure 1).

### 3.2. Breast Cancer

Breast cancer (BC) is the most common tumor affecting women worldwide. Despite local and adjuvant therapy, bone metastases appear in 70% of cases, impacting negatively on patient survival and quality of life [21].

The most widely used imaging modality to detect bone metastasis in breast cancer is BS and PET/CT with (18)F-NaF, but several studies demonstrate the limited sensitivity and specificity of these techniques [22].

WB-MRI with and without DWI seems to be effective in the assessment of bone marrow metastases in patients with advanced BC, but it is important to determine the most useful protocol in these patients.

Grankvist et al. demonstrated that the T1W sequence had the best sensitivity (98%) but low specificity (77%) in the detection of breast metastases. However, with the addition of STIR and DWI, the specificity increased to 95%. The sensitivity of DWI was 70% [23]. The authors concluded that MRI with T1, STIR, and DWI is useful for the evaluation of secondary bone lesions from BC and compares well to PET/CT.

In the study by Heusner et al., who compared DWI alone with PET/CT, DWI demonstrated a lower sensitivity (86%) in bone metastases detection than PET/CT (100%), which could be explained by DWI’s low spatial resolution and the susceptibility of DWI to artifacts [24].

Moreover, in a study by Pearce et al. who compared bone metastases detection from BC, PC, and myeloma, it was reported that DWI conspicuity is similar to that of the STIR sequence in BC. However, it is higher in the other two tumors. The authors concluded that this difference can be determined by the different cellular microstructure of the neoplasm (metastases from the breast are mixed, i.e., sclerotic/lytic) [25].

Jambor et al., in their region-based analysis, found that the WB-MRI protocol, including T1W, STIR, and DWI sequences, has a sensitivity of 91%, a specificity of 99%, and an accuracy of 97%. They also reported that DWI associated with STIR and T1W images can reduce false positive lesions [22].

**Figure 2:**
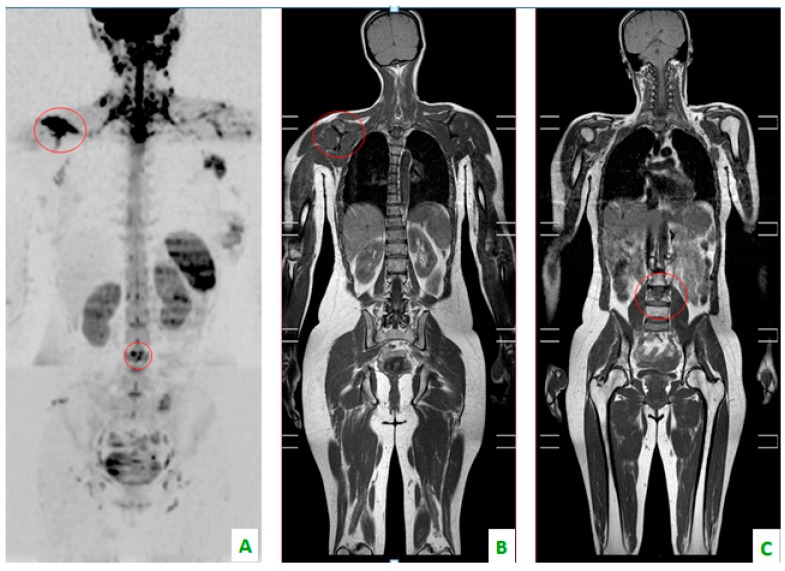
Bone localizations of breast cancer in the coronal plane: scapula and vertebral metastases (in the red circles). (**A**) DWI with a “positron emission tomography (PET)-like” view. The right scapula and one lumbar vertebra present an alteration of diffusivity. (**B**) T1 DIXON showing the hypointensity of the scapula and (**C**) lumbar soma.

Moreover, like in PC bone metastases, quantitative evaluation using DWI can be useful for monitoring therapy response and for the early detection of non-responders, in order to change their therapy [22] (Figure 2).

### 3.3. Lung Cancer

In non-small cell lung cancer (LC), secondary lesions commonly localize in bone. Bone metastasis detection is not only important in determining the stage of the disease but also in reducing the comorbidity associated with a worse quality of life [26].

**Figure 3:**
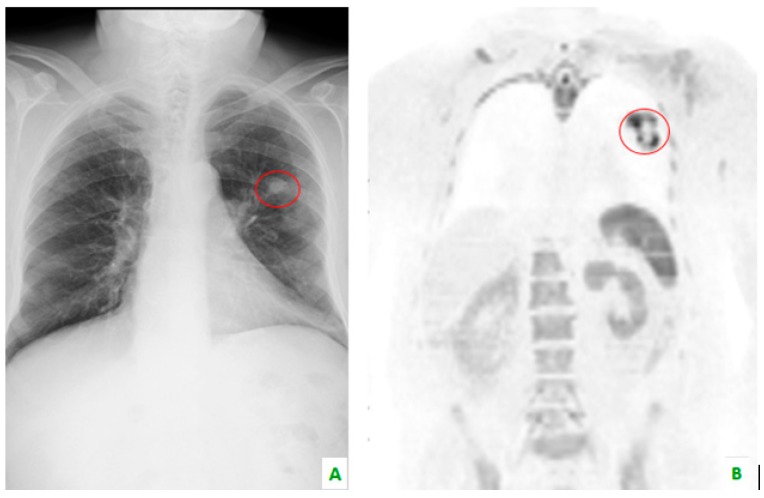
(**A**) This chest X-ray reveals, in the red circle, a lung tumor which is also visible in (**B**) whole body magnetic resonance imaging with diffusion-weighted imaging (WB-MRI-DWI) in the coronal plane, in the red circle.

**Figure 4:**
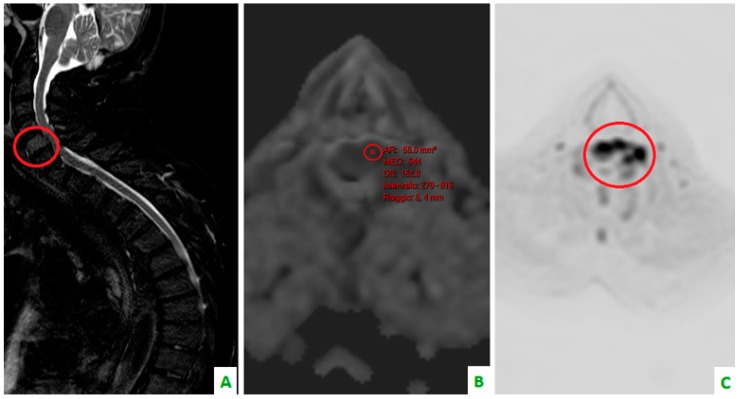
Focus on the red circles: (**A**) DIXON T2 weighted in the sagittal plane showing bone cervical metastasis (C5) (**B**) a low apparent diffusion coefficient (ADC) value in the same level, and (**C**) a restriction of diffusivity.

**Figure 5:**
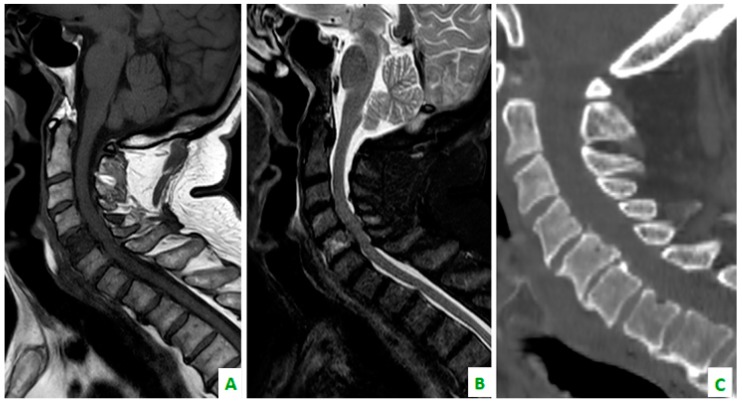
(**A**) A T1 sequence and (**B**) STIR sequence, performed with a dedicated spinal MRI, highlight the lung metastases. (**C**) Cervical spine on a sagittal reconstruction of a computed tomography (CT). The bone metastasis is not so clear.

Skeletal metastases, which are predominantly osteolytic, localize in the spine in 50% of patients. This the most common area of localization, followed by the ribs (27.1%), ilium (10%), sacrum (7.1%), femur (5.7%), and the humerus, scapula, and sternum (2.9%). This is according to a retrospective study by Tsuya et al. [27] (Figure 3, Figure 4 and Figure 5).

BS and PET/CT, used to detect the neoplasia cited above, remain the most commonly employed techniques for suspected bone metastases caused by LC. Unfortunately, comparative studies of WB-MRI-DWI with other techniques are very few. Most of the studies focused on the comparison of WB-MRI standard protocol with PET/CT and BS [28,29].

A study performed by Takenaka et al. assessed the utility of DWI by analyzing 115 patients with non-small cell lung carcinoma, demonstrating that WB-MRI detected bone localization with more sensitivity than BS or BS integrated with PET/CT and more specificity than DWI alone. Association with DWI sequences and WB-MRI improves the specificity and accuracy of WB-MRI [30].

A study by Ohno et al. concluded that the ability of DWI alone in the detection of metastases, including bone localizations, was lower than WB-MRI with DWI imaging and integrated with PET/CT. The same results were obtained with regards to specificity and accuracy [31].

In conclusion, nowadays, WB-MRI-DWI and PET/CT are the most sensible techniques to identify bone metastasis in LC as well.

### 3.4. Thyroid Cancer and Melanoma

Bone metastases are complications of thyroid carcinoma, especially of the follicular type, which determine a significant reduction of quality of life by causing pain and pathological fractures. Patients with differentiated thyroid carcinoma (DTC) have a 10-year survival rate of 80–95%. However, when distant metastases are present, the overall 10-year survival rate reduced to 40% [32].

Unfortunately, in literature, reports about WB-MRI-DWI detection of bone metastases due to thyroid cancer are limited.

One of them, the study by Sakurai et al., examined 23 patients with differentiated thyroid carcinoma and total thyroidectomy who underwent WB-MRI with standard protocol, WB-MRI with DWI, and WB PET/CT. Authors showed that the sensitivity of WB-MRI with DWI in detecting bone metastases was higher (82%) compared to WB-MRI without DWI (64%) and PET/CT (79%). The techniques had an WB-MRI with DWI accuracy of 94%, 90%, and 94%, respectively. Authors concluded that DWI improves the sensitivity and accuracy of WB-MRI in the identification of secondary skeletal lesions in patients with DTC [33].

In another work by Nagamachi et al., the authors evaluated 70 postoperative DTC patients who underwent whole body scintigraphy, PET/CT, and WB-MRI-DWI. The study demonstrated that the detectability of the three techniques was 67.1%, 84.2%, and 57.6%, respectively. Authors concluded that DWI could be the method of choice for monitoring postoperative DTC for possible secondary lesions. In this study WB-MRI alone, without a DWI sequence, was not evaluated [34].

Bone is a frequent site for metastases in patients with malignant melanoma, detectable in about 23–49% of cases [35]. Some reports have tried to determine the role of WB-MRI-DWI, but this technique is used more often for the loco regional staging of melanoma.

Mosavi et al. found that the sensitivity of WB-MRI and DWI varies considerably in different regions of the body. At the level of the bone, WB-MRI and WB-MRI-DWI detected a significantly higher number of bone lesions compared to CT. WB-MRI and DWI equally detected 56 bone lesions in 12 patients, while CT showed 42 bone lesions in 8 patients [36].

Laurent et al. compared WB-MRI, including DWI, and PET/CT and demonstrated a higher sensitivity and specificity for WB-MRI in the detection of melanoma bone metastases. The sensitivity and specificity for WB-MRI were 82% and 97%, respectively, and 72.8% and 92.7% for PET/CT, respectively. DWI detected 20% more lesions compared with the standard MRI protocol [37].

Petraglia et al. compared WB-MRI-DWI with contrast-enhancement (CE MRI) in detecting bone metastases in patients with advanced melanoma. The authors concluded that WB-MRI-DWI without additional CE MRI sequences is promising for the detection of extracranial metastases in melanoma patients, but CE MRI is necessary for evaluating the brain [38].

DWI is able to detect bone metastases in patients with thyroid carcinoma and malignant melanoma with a higher sensitivity and specificity compared to WB-MRI standard protocol, PET/CT, and bone scintigraphy.

## 4. Conclusions

WB-MRI-DWI seems to be a promising method of imaging in the detection of bone metastases. Standardization of protocols and more studies are needed (Table 1).

## Figures and Tables

**Table 1:** Summary of some articles about nuclear medicine and WB-MRI-DWI cited in the text.

Article (First Author, Year)	Imaging Methods	Patients	Results	Conclusions
M.A. Jacobs et al., 2018	- Multiparametric WB-MRI-DWI (3 T) - Standard: computed tomography (CT) and PET	54	WB-MRI: excellent sensitivity (96%) ADC map values were significantly increased (*p *< 0.05) compared to normal bone.	Multiparametric WB-MRI feasible for oncologic staging to identify bone metastasis
H. Wieder et al., 2017	- ^11^C-choline PET/CT - MRI (T1 + STIR + DWI) - Standard: follow-up	50	PET/CT was significantly superior to MRI in detecting bone metastasis (*p* = 0.02).	^11^C-choline PET/CT superior than WB-MRI-DWI
L.L. Peng et al., 2015	Metanalysis: - WB-MRI-DWI (1.5–3T) - BS - PET/CT	1507	per-patient basis: sensitivity 95% specificity 92% per-lesions: sensivity 92% specificity 95%	Studies support DWI in detection of bone metastasis from different tumors
I. Jambor et al., 2015	- ^99m^Tc-HDP BS - ^99m^Tc-HDP SPECT - ^99m^Tc-HDP SPECT/CT - ^18^F-NaF PET/CT - 1.5 WB MRI DWI - Standard: clinical/radiological consensus and f-up	53	sensitivity values: 62% ^99m^Tc-HDP BS 74% ^99m^Tc-HDP SPECT 85% ^99m^Tc-HDP SPECT/CT 93% ^18^F-NaF PET/CT 91% 1.5 WB-MRI including DWI	^18^F-NaF PET/CT and 1.5 WB-MRI including DWI had similar accuracy
F. Mosavi et al., 2012	- ^18^F-NaF PET/CT - 1.5 WB-MRI-DWI - Standard: BS, conventional MR images, and follow-up	49	18F-NaF PET/CT vs. DWI, *p* = 0.27 DWI vs. reference, *p* = 0.64 ^18^F-NaF PET/CT vs. reference, *p* = 0.06	- ^18^F-NaF PET/CT high sensitivity - Whole-body DWI higher specificity but lower sensitivity than ^18^F-NaF PET/CT
Grankvist et al., 2012	- 3 T WB-MRI-DWI - ^18F^FDG-PET/CT	13	T1: sensitivity 98%, specificity 77%, T1 + STIR + DWI: specificity 95%	T1 + STIR + DWI is useful to detect bone metastasis
S. Nagamachi et al., 2011	- ^131^IWBS - ^18F^FDG-PET/CT - DWI	70	BS (18)F-FDG PET/CT and WB DWI demonstrated that detectability of three techniques was 67.1%, 84.2%, and 57.6%	IWBS and DWI might be the method of choice in follow of post operative differentiated thyroid cancer
D. Takenaka et al., 2009	- DWI - MRI without and with DWI - ^18F^FDG-PET/CT - Standard: BS, FDG-PET/CT, WB MR	115	In lesions based analysis: accuracy of WB-MRI-DWI + FDG-PET/CT > WB-MRI without DWI (*p *< 0.05) accuracy of WB-MRI-DWI > BS + FDG-PET/CT (*p *< 0.05) In patients based analysis: accuracy of WB-MRI-DWI > BS (*p *< 0.05)	WB-MRI with DWI similar accuracy of bone scintigraphy and/or PET/CT to detect bone metastasis in NSCLC
C.A. Yi et al., 2008	- PET/CT - 3.0 Tesla WB-MRI - standard: follow up and biopsies	165	PET/CT and whole-body MR imaging, metastatic bone metastasis were detected in 67% patients	WB-MRI:higher sensitivity than BS lower sensitivity than FDG PET.
